# Gray’s Anatomy. Spitzka Edition

**Published:** 1914-03-15

**Authors:** 


GRAY'S ANATOMY. SPITZKA EDITION. That a new edition of Gray's Anatomy was needed by the dental profession is scarcely to be expected, for the reason that we study our Anatomy largely from special text books. But there are times when we need to know our Anatomy as we learned it in College and have to go back and blow the dust off of our old Gray to refresh our memories. Some dentists would be surprised to know that there has been any progress made in Anatomical Knowledge. We at least don't see how there can be any more necessity for revising Gray's Anatomy than there is for revising the family bible, or the Ten Commandments. Nevertheless if they were to take a look at the new Anatomy recently published by Lea & Febiger, and completely revised and brought down to date they would be able to recognize the fact that whilst they had slept great strides in Anatomical progress had been made. Students and teachers of Anatomy will rejoice in this new work for it is not only modern in facts of Anatomy, but the text and illustrations are so complete that the students' needs are completely met, and much new material that will stimulate desire for more accurate Anatomical Knowledge ought to be of great value to practitioners who wish to keep up to date in modern surgical procedures. The editor, Professor E. A. Spitzka, of Jefferson Medical College has added materially to the text, and the illustrations. Many of them new and in colors makes the work complete and all that we can desire.

